# Trends and age-period-cohort effect on dental caries prevalence from 2008 to 2019 among Brazilian preschoolers

**DOI:** 10.1590/1807-3107bor-2024.vol38.0004

**Published:** 2024-01-05

**Authors:** Yassmín Hêllwaht RAMADAN, Jessica Klöckner KNORST, Bruna BRONDANI, Bernardo Antonio AGOSTINI, Thiago Machado ARDENGHI

**Affiliations:** (a) Universidade Federal de Santa Maria – UFSM, School of Dentistry, Department of Stomatology, Santa Maria, RS, Brazil.; (b) Universidade de São Paulo – USP, School of Dentistry, Department of Pediatric Dentistry and Orthodontics, São Paulo, SP, Brazil.; (c) Atitus Educação, Graduate Program in Dentistry, Passo Fundo, RS, Brazil.

**Keywords:** Child, Dental Caries, Prevalence, Time Factors

## Abstract

This study aimed to evaluate trends in the prevalence of dental caries in preschool children and associated factors considering different time variations. This is a time series study performed using data from three cross-sectional studies with pre-school children from southern Brazil in 2008, 2013 and 2019. This children group was born between the years of 2003 to 2018. Dental caries was evaluated by decayed, missing and filled deciduous teeth (dmft index). Demographic, socioeconomic, behavioural and psychosocial variables were also collected. Chi-square test for trends and a hierarchical age-period-cohort (HAPC) analysis using multilevel Poisson regression model for testing the associations between predictor variables and dental caries experience were used. A total of 1,644 pre-school children participated in all surveys. There was a significant difference in caries experience considering all APC effects. The prevalence of dental caries was 25.0% in 2008, 16.3% in 2013, and 19.4% in 2019 (p < 0.01) and no statistical difference was observed. An age effect showed that older children were more likely to experience dental caries. Considering the cohort effect, there is a significant difference between the generations, mainly between 2003 and 2018. Household income, use of dental services, and parent’s perception of child oral health were associated with dental caries experience no matter the time variation. Despite recent declines in dental caries prevalence among preschool children, caries levels increased with age and social inequalities persisted through the years, indicating a need of reviewing the policies to reduce the burden of this oral disease.

## Introduction

Despite the decline in global rates in recent decades, dental caries is still one of the most prevalent chronic diseases. Data from the last Global Burden of Disease Study showed that there was a 1.15 billion increase in the number of new cases of untreated dental caries in deciduous teeth between the years 1990 and 2019. Of these, about 62.9 million can be attributed to sociodemographic inequalites.^1^ Dental caries not only negatively impacts the physical and social well-being of affected individuals,^2^ but also has a high economic impact,^3^ reflecting widespread social and economic inequalities, particularly in low- and middle-income countries.^4^ It is estimated that in 2015, the indirect cost due to caries in deciduous teeth was $0.90 billion.^3^ Thus, dental caries is still considered a public health problem and a relevant oral health outcome, especially when it occurs in early life.

The risk factors for dental caries vary according to individual and environmental factors.^5-9^ Previous studies have shown that individuals with socioeconomic disadvantages have higher levels of dental caries.^5,6^ Poor hygiene, dietary habits, characteristics related to social capital, and place of living have also been associated with more burden of the disease.^6-8^ Furthermore, a gradient in the occurrence of dental caries with increasing age has been observed.^9^ Thus, there is a need to monitor the changes and trends in the prevalence of dental caries considering chronological age and different time periods.

A possible way of evaluating this aspect is using a time series, where three types of time-related variations can be considered: age, period, and cohort (APC) effects. Understanding the impact of each effect is important for assessing individual and socially related predictors for inequalities in oral health outcomes over time.^10,11^ Age effects refer to variations associated with different age groups, representing the developmental changes that occur during life. Period effects refer to variations in the calendar years that affect all age groups simultaneously and are related to cultural and economic changes over a specific time. Cohort effects refer to variations among groups born in different years, representing the effects of formative experiences and early life exposures related to social and generational changes.^10,11^

Although some studies have evaluated the detailed analysis of changes in dental caries levels considering the APC effects, they were related to permanent dentition^12,13^ or were performed more than 3 decades ago.^14^ Thus, it is important to know the current trend of the disease in primary dentition in order to plan more effective prevention measures, especially for members of social minorities, reducing dental caries inequities. This aspect is particularly important in early childhood, once caries experience at this phase is a strong predictor of dental caries throughout the life course.^9^ Thus, this study aimed to verify trends in the prevalence of dental caries in preschool children and associated factors considering the age-period-cohort effects.

## Methodology

### Study design and sample

This is a trend study that used data from three cross-sectional studies performed in 2008, 2013, and 2019 with pre-school children (from 0 to 5 years old) in Santa Maria, a medium-size city in southern Brazil. This municipality has an estimated population of 285,159, of which approximately 28,000 are under the age of 6 years, according to the most recent populational census.^15^ The Human Development Index of Santa Maria is 0.845, which is higher than the national average (0.761). The city has a fluoridated water supply.

All surveys were carried out on National Children’s Vaccination Day. Preschoolers were selected systematically through health units with a dental chair, being eight in 2008, and 15 in both 2013 and 2019. The health units were distributed in different neighborhoods of the city and included about 90% of the children vaccinated in the municipally. For recruitment, every fifth child in line for vaccination was invited to participate in the study. If caregivers did not authorize their participation, the next child in line was selected. Children who presented disability with cognitive impairment were excluded from the sample.

The sample size calculation considered a sampling error of 5%, confidence level of 95%, estimated population of preschoolers in the municipally (28,000), and the expected maximum caries prevalence of 50%. Considering a design effect of 1.2 and adding 10% for possible refusals, the minimum required sample size was 501 individuals in each year.

### Data collection and variables

Data collection in all periods was performed through clinical examinations, interviews, and structured questionnaires following standardized criteria for oral health surveys.^16^ The research team consisted of 15 calibrated examiners plus 30 trained assistants for recruitment of the pre-school children and questionnaire application.

Dental caries was evaluated by decayed, missing, and filled deciduous teeth index (dmft index).^16^ Children were examined in a private room with a dental chair in the healthcare centers. Dental exams were performed using a flat dental mirror and CPI “ball point” periodontal probes. In all surveys, about fifteen examiners were previously trained and calibrated following the WHO recommendations, totaling 36 hours. The process first involved a theoretical class ministered by a gold-standard researcher, followed by an evaluation of photographs and a discussion of cases. Subsequently, a clinical-epidemiological exercise was performed using 10 exfoliated primary teeth. In 2008, inter- and intra-examiner kappa coefficients for the dmft ranged from 0.77 to 0.95 and from 0.80 to 0.94, respectively. In 2013, inter- and intra-examiner kappa coefficients ranged from 0.70 to 0.95 and from 0.73 to 0.88, and in 2019, both coefficients ranged from 0.70 to 1.00. For data analysis, caries experience was considered as absent (dmft = 0) or present (dmft > 1).

Questions were also asked about dental visits and parents’ perception of the child’s oral health. Use of dental services was evaluated using the following question: “In the last year (12 months), how many times have you been to the dentist?”, proposed by WHO.^16^ Preschoolers were dichotomized to whether they had visited the dentist at least once in the last year (regular users) or not (non-regular users).^16^ Parents’ perception of their child’s oral health was assessed with the question: “Would you say that the health of your child’s teeth, lips, jaws, and mouth is…,” and response options (0) “excellent,” (1) “very good,” (2) “good,” (3) “fair,” and (4) “poor”. For data analysis, the variable was dichotomized into excellent or good (0,1 and 2) and fair or poor (3 or 4).^17^

Some demographic and socioeconomic variables were also collected to explain the distribution of dental caries. Demographic variables included sex (boys or girls), age (in years), and skin color, evaluated using the criteria established by the IBGE^15^ and later dichotomized into white and non-white. The socioeconomic level was assessed through monthly household income and maternal education. Household income was collected in real (Brazilian currency [R$] – the exchange rate is approximately US$1.00 to R$5.40) and subsequently categorized by the median (R$ 1,390). Maternal education was collected in years and posteriorly dichotomized into ≥ 8 years or < 8 years of formal education (incomplete elementary education).

### Ethical issues

All surveys used in this study were previously approved by the Research Ethics Committee of the Federal University of Santa Maria (protocol number 2008: 0090.0.243.000-08; protocol number 2013: 18512213.5.0000.5306; and protocol number 2019: 18426219.5.0000.5346). All children agreed to participate in the study and their caregivers signed an informed consent form.

### Data analysis

Data were analyzed using STATA 14.0 statistical software (StataCorp. 2014. Stata Statistical Software: Release 14.0. College Station, USA: StataCorp L). A descriptive analysis of sample characteristics was performed. The data analysis accounted for the age-period-cohort effect. Four age groups (chronological age): 1, 2-3, 4, and 5 years, three evaluation period groups (year of examination): 2008, 2013, and 2019, and twelve cohort groups (year of child’s birth), from 2003 to 2018, were considered. A chi-square test for trends was used to compare dental caries experience according to the APC effects.

Multilevel Poisson regression analysis considering the hierarchical age-period-cohort (HAPC) model for evaluating the association between sample characteristics and dental caries experience was also performed. HAPC treats periods and cohorts at the contextual level and age as individual characteristics.^18^ This model was executed considering random effects. Variables with p ≤ 0.20 in the unadjusted analysis were eligible for inclusion in the adjusted analysis. The variables were included in the adjusted model using the forward method. Maternal education was removed from the final model due to high collinearity with household income. Results are presented as prevalence ratio (PR) and a 95% confidence interval (95%CI).

## RESULTS

Of the 1,671 individuals included in all epidemiological surveys, 580, 546, and 545 were evaluated in 2008, 2013, and 2019, respectively. The sample was balanced between boys and girls in all surveys. The mean age was 3.1 (standard deviation [1.6]) in 2008, 3.3 (SD 1.6) in 2013, and 3.3 (SD 1.6) in 2019. Most individuals had white skin and most mothers had 8 years or more of formal education. Most preschoolers had not attended dental services in the previous year. The dmft means (SD were of 0.8 (SD 2.0), 0.5 (SD 1.6), and 0.6 (SD1.7) in 2008, 2013, and 2019, respectively. More details about sample characteristics are shown in Table 1.

**Table 1:** Descriptive characteristics of the sample according to survey year - Santa Maria, Brazil (n = 1,671)

Variables	n (%)	n (%)	n (%)	n (%)*

	2008	2013	2019	Total
Sex				
Boys	309 (53.3)	283 (51.8)	282 (51.7)	874 (52.3)
Girls	271 (46.7)	263 (48.2)	263 (48.3)	797 (47.7)
Age (years)				
1	132 (22.9)	118 (21.7)	107 (20.6)	357 (21.7)
2–3	127 (21.9)	106 (19.4)	112 (21.5)	345 (21.0)
4	146 (25.2)	146 (26.8)	121 (23.3)	413 (25.1)
5	174 (30.0)	175 (32.1)	180 (34.6)	529 (32.2)
Skin color				
White	448 (77.2)	441 (81.2)	396 (73.3)	1,285 (77.3)
Non-white	132 (22.8)	102 (18.8)	144 (26.7)	378 (22.7)
Household income in reals (R$)				
< R$ 1,390	296 (52.2)	264 (49.1)	244 (49.1)	804 (50.4)
> R$ 1,390	271 (47.8)	266 (50.2)	253 (50.9)	790 (49.6)
Maternal education (years)				
> 8	394 (69.5)	423 (78.8)	453 (86.0)	1,270 (77.9)
< 8	173 (30.5)	114 (21.2)	74 (14.0)	361 (22.1)
Dental attendance in the last year				
Yes	138 (23.8)	135 (25.1)	163 (30.8)	436 (26.5)
No	441 (76.2)	404 (74.9)	367 (69.2)	1,212 (73.5)
Parents’ perception of their child’s oral health				
Excellent or good	470 (81.1)	446 (82.0)	332 (75.3)	1,248 (79.8)
Fair or poor	109 (18.9)	98 (18.0)	109 (24.7)	316 (20.2)
dmft > 1				
Not	435 (75.0)	457 (83.7)	439 (80.6)	1,331 (79.7)
Yes	145 (25.0)	89 (16.3)	106 (19.4)	340 (20.3)
dmft [mean (SD)]	0.8 (2.0)	0.5 (1.6)	0.6 (1.7)	0.7 (1.8)

R$: Real (exchange rate is US$1.00 to R$5.4, approximately); dmft: decayed, missing and filled deciduous teeth; SD: standard deviation. *Values lesser than 1,671 are due to missing data.

Figure displays the trends in prevalence of caries experience according to the APC effect. There was a significant difference in caries experience through the years considering all APC effects. The prevalence of preschool children who experienced dental caries was 25.0% in 2008, 16.3% in 2013, and 19.4% in 2019 (p < 0.01). An age effect showed that older children were more likely to experience dental caries (p < 0.01). The cohort effect varied nonlinearly over the years, but there was a significant difference in caries prevalence among generations (p <0.01).

**Figure:**
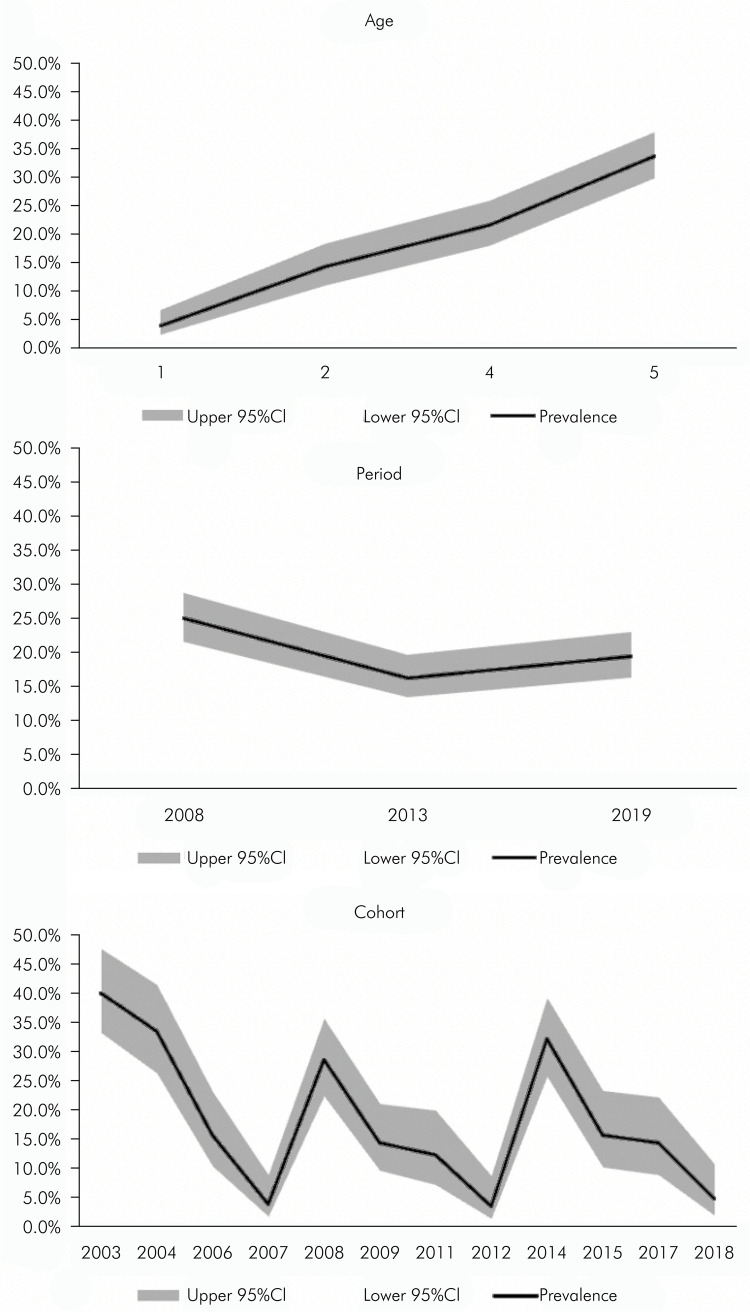
Trends in prevalence of dental caries experience (dmft ≥1) by age, period, and cohort (n = 1,644).

Multilevel Poisson regression analyses in dental caries prevalence and associated factors considering the APC effects are presented in Table 2. In the unadjusted analysis, age, skin color, household income, maternal education, use of dental services, and parents’ perception of their child’s oral health were associated with dental caries experience in the surveys. In the adjusted analysis, there was a significant association of chronological age so that 5-year-olds had 6.1 times higher prevalence of dental caries than 1-year-olds (PR 6.10; 95%CI 3.32–11.21). Preschoolers whose household income was lower than R$1,390 presented a 27% higher prevalence of dental caries than their counterparts (PR 1.27; 95%CI 1.01–1.61). Individuals who did not use dental services in the previous year were protected from experiencing dental caries (PR 0.63; 95%CI 0.50–0.80). In addition, parents’ perception of their child’s oral health as fair or poor was associated to a 2.35-fold higher prevalence of dental caries (PR 2.35; 95%CI; 1.86–2.98).

**Table 2:** Unadjusted and adjusted multilevel Poisson regression analysis in dental caries prevalence and associated factors considering age-period-cohort (n = 1,644).

Variables	Unadjusted	p-value	Adjusted^#^	p-value
	
	PR (95% CI)		PR (95% CI)	
Sex				
Boys	1.00		1.00	
Girls	0.96 (0.77–1.19)	0.732	0.98 (0.78–1.23)	0.893
Age (years)				
1	1.00		1.00	
2–3	3.51 (1.94–6.38)	< 0.01	3.21 (1.69–6.10)	< 0.01
4	5.50 (2.84–2.97)	< 0.01	4.45 (2.40–8.24)	< 0.01
5	7.73 (4.40–13.56)	< 0.01	6.10 (3.32–11.21)	< 0.01
Skin color				
White	1.00		1.00	
Non-white	1.34 (1.05–1.71)	< 0.05	1.18 (0.91–0.154)	0.188
Household income in Reals (R$)				
< 1,390	1.00		1.00	
> 1,390	1.42 (1.13–1.78)	< 0.01	1.27 (1.01–1.61)	< 0.05
Maternal education (years)				
> 8	1.00			
< 8	1.62 (1.27–2.06)	< 0.01		
Dental attendance in the last year				
Yes	1.00		1.00	
No	0.66 (0.53–0.83)	< 0.01	0.63 (0.50–0.80)	< 0.01
Parents’ perception of their child’s oral health				
Excellent or good	1.00		1.00	
Fair or poor	2.55 (2.04– 3.19)	< 0.01	2.35 (1.86–2.98)	< 0.01

R$: Real (US$1.00 is equivalent to R$5.4 approximately); PR: prevalence ratio; CI: confidence interval. ^#^Adjusted by cohort and period at the second (contextual) level and by the other variables in the model at the first (individual) level.

## Discussion

This study aimed to evaluate trends in dental caries prevalence in preschool children and associated factors accounting for age-period-cohort effects. Our findings showed a significant change in dental caries prevalence according to different time variation of age, period, and cohort. Although previous studies have evaluated the prevalence of dental caries considering APC effects,^12-14^ the assessment of these factors in preschoolers in the last two decades had not been explored yet. Moreover, household income, use of dental services, and parents’ perception of child oral health were also associated with dental caries experience, even in the presence of these time variations effects. This highlights the influence of socioeconomic and behavioral factors in caries occurrence.

Age affected the occurrence of caries, indicating that older children have a greater dental caries experience. It is well stablished in the literature that there is a notable gradient in caries occurrence as age increases.^9^ Our study found similar results, indicating that 5-year-olds are about six times more likely to have caries when compared to 1-year-olds. A possible explanation for this finding is the higher number of teeth exposed to risk factors according to eruption chronology and age of such exposure. Further, dental caries is a cumulative disease and the dmft index measures past and present caries experience.^12^ Thus, longer exposure of teeth to risk factors over the years and the cumulative effect of oral problems, as well as harmful eating habits, such as increased consumption of cariogenic foods^19-20^ may lead to higher levels of dental caries with increasing age.

Over the years analyzed, overall caries prevalence decreased between 2008 and 2013 and had a slight, non-significant, increase in the last period (2019) but not significantly. A recent systematic review has shown that in the last two decades there have been no significant improvements in the prevalence of untreated dental caries in the primary dentition, some stability maintained.^1^ In addition, in Latin American and Caribbean countries, although caries prevalence has been reducing, it is still considered high, being around 55% for the primary dentition.^21^ Another possible explanation for the increase in caries prevalence is the worsening of economic indices that occurred before the last period. Between 2015 and 2016, Brazil faced one of the five biggest economic crises in history.^22^ Since the occurrence of dental caries in preschoolers in 2019 is the result of worse conditions in the first years of life, lower income, worse housing conditions, and parental unemployment may result in higher levels of disease over time.^6,23^Furthermore, the lower dental caries prevalence in 2013 and its increase in 2019 may also be explained by the country’s political context. Especially between 2017 and 2019, federal funding for oral health was reduced, changing the national oral health policy and consequently decreasing the level of actions carried out in the country.^24,25^ This scenario may have affected the higher prevalence of the disease in 2019.

Our findings also demonstrated the cohort effects, which refer to variations among groups born in different years. A significant difference was found between the cohorts, but without a clear and linear progression or regression. Based on the entire distribution of the prevalence we could suggest that younger cohorts presented lower levels of dental caries. This result is in agreement with the most recent evidence, showing that, globally, dental caries rates in younger age groups have declined over the last few decades.^1,21^ This may be due to the use of preventive methods, such as the widespread water fluoridation and used of fluoridated toothpastes, as well as changes in the social and economic characteristics of the population.^21^ Despite that, dental caries reduction has not occurred equally in the population, and non-white children, whose mothers usually present lower education and income, remain with a higher burden of the disease, which reflects a global polarization of dental caries, especially in middle- and low-income countries.^4,26^ Still considering the year of birth, children born before 2010 belong to ‘Generation Z’, while children born after 2010 belong to ‘Generation Alpha’. This change may have implications for increased access to screens and changes in family structure, which in turn may impact oral health. In this context, future studies evaluating factors related to this generational transition are suggested.

Our findings also showed that preschoolers from families with lower income were more likely to present dental caries than their counterparts even when considering time variations. In the present study, we considered household income as a proxy to individual socioeconomic status, and family income has been associated with several oral health outcomes and dental caries in the primary dentition in previous studies^27,28^. Individuals from lower socioeconomic backgrounds are more exposed to several risk factors that can affect oral health^29^, as they usually live in worse housing conditions and present poorer health behaviors, such as less access to dental services and poor oral hygiene habits^17,30^. Thus, our study corroborates the knowledge that socioeconomic conditions leads to worse oral health conditions, such as a higher prevalence of dental caries.

Individuals who did not use dental services in the previous year before data collection were protected from experiencing dental caries. A previous study showed that children who have higher dental caries rates visited the dentist more frequently than their counterparts.^31^ Thus, it is hypothesized that individuals with more dental caries experience more pain and difficulties in daily life,^2,32^ leading to greater utilization of dental services. This result shows that dental services are still mainly used for curative treatments and approaches focusing on changes towards preventive dental care are extremely important. The cost-effectiveness of a preventive approach to oral health has been shown to be less costly and more effective in this age group,^33^ resulting in lower economic costs to public health in the country. Furthermore, our findings indicate that parent’s perception of their child’s oral health as fair or poor was related to a higher dental caries prevalence, in agreement with previous studies.^6,34^ It has been shown that parents who negatively perceive their child’s oral health status may be less likely to care for their children’s oral health hygiene, which may be related to a higher dental caries experience.^34^

This study has some limitations that need to be considered. First, the data analysis included only three time points, which does not allow to trace all time effects, especially for period-effect. However, our trend study evaluated deciduous teeth in a period of 10 years, so well defined pattern in disease occurrence in this population could be established. Furthermore, this is a time-series study, and such a design does not allow us to trace cause-effect relationships; longitudinal studies are recommended for this purpose. However, the same strategy has been used in other studies and provides us substantial information about behavior populational in a specific disease^12,35^Another limitation that needs to be described is about the potential confounding factors related to the APC methodology. This type of analysis aims to describe and estimate the independent effect of age, period, and cohort on the outcome by partitioning the variance into single components.^36^ However, due to the exact linear dependence between age, period, and cohort, it is not possible to independently estimate each of these effects through data modeling, which is known as the “identification problem” in APC. To overcome this problem, alternatives have been developed, such as the hierarchical age-period-cohort (HAPC),^18,37^ which was used in this study and supports the validity of our findings.

Some strengths of the study should also be highlighted. First, our data are from a representative sample of preschoolers in the city and included neighborhoods from different socioeconomic backgrounds, which provides external validity to our findings. In addition, the sample points used for data collection covered about 90% of the children vaccinated in the municipality in each survey period. Finally, it should be noted that our study considered the APC effects during early childhood. Understanding the pattern of dental caries occurrence and the predisposing factors in this period is essential to preventing poor oral problems throughout the life course.

## Conclusion

Our findings demonstrated the impact of age-period-cohort effects on the pattern of dental caries occurrence. In general, a significant reduction in dental caries prevalence was found over periods-cohorts and an increase according to age. Household income, use of dental services, and parents’ perception of child oral health were associated with dental caries experience. Despite the recent decline in dental caries prevalence among preschool children, caries increased with age and social inequalities persisted through the years, indicating that public policies to reduce the burden of this oral disease must be improved.
